# SKA1 promotes tumor metastasis via SAFB-mediated transcription repression of DUSP6 in clear cell renal cell carcinoma

**DOI:** 10.18632/aging.204418

**Published:** 2022-12-02

**Authors:** Yan Pu, Jing Han, Mengmeng Zhang, Mengxue Liu, Gulnazar Abdusamat, Huibin Liu

**Affiliations:** 1 Institute of Cancer Research, Affiliated Tumor Hospital of Xinjiang Medical University, Urumqi 830011, PR China; 2 Department of Pharmacy, Xinjiang Medical University, Urumqi 830011, PR China; 3 The Clinical Research Center of Breast Tumor and Thyroid Tumor in Xinjiang Autonomous Region, Urumqi 830011, PR China

**Keywords:** SKA1, ccRCC, SAFB, DUSP6, metastasis

## Abstract

The most hostile form of urologic cancer, clear cell renal cell carcinoma (ccRCC), has a high fatality rate and poor prognosis due to tumor metastasis at initial presentation. The complex process driving ccRCC metastasis is still unknown, though. In this study, we demonstrate that Spindle and kinetochore-associated protein 1 (SKA1) expression is significantly upregulated in ccRCC tissues and associated with aggressive clinicopathologic characteristics. Functionally, SKA1 knockdown on ccRCC cells reduced cancer cell motility both *in vivo* and *in vitro* research. These bioactivities of SKA1 may be brought on by its specific interaction with scaffold attachment factor B, according to the proposed mechanism (SAFB), which could further depress the transcription of dual specificity phosphatase 6 (DUSP6). Our findings may provide a new way of researching SKA1-regulated tumor metastasis, and indicate that SKA1 is a prospective therapeutic target for renal carcinoma.

## INTRODUCTION

The term “renal cell carcinoma” (RCC) refers to a diverse range of histologic abnormalities, the most common subtype of which is clear cell renal cell carcinoma (ccRCC) [1]. The aggressive behavior is due to its potential for metastatic diffuse and 30% of cases are metastatic at the time of presentation [2]. Over the past decade, multiple targeted therapies and immunotherapy have dramatically improved the overall survival (OS) rate of patients [3–5]. However, in the great majority of individuals with metastatic renal cell carcinoma, long-lasting effectiveness is infrequent (mRCC) [6]. So far, we know little about the molecular events that facilitate cancer progression, and a better understanding of the biological basis of disease will ensure dramatic advances in system therapy in the metastatic setting. In the human genome, spindle and kinetochore-associated protein 1 (SKA1), together with SKA2, SKA3 constitute the SKA complex; this is necessary to maintain spindle microtubule attachment to kinetochores during mitosis [7–9]. Recent studies reveal the involvement of SKA1 in the growth and proliferation of numerous cancer types. SKA1 has garnered considerable interest in the field of malignancies, and it has been implicated in the development and spread of many cancer types [10–14]. Although it is known that SKA1 is overexpressed in RCC, its role in kidney cancer progression and metastasis remains unclear. In addition, little is understood about the potential processes and downstream targets of SKA1 in tumor metastasis.

Here, using models both *in vitro* and *in vivo*, we have corroborated that SKA1 is functional as an oncogenic molecule in ccRCC and have, for the first time, demonstrated that SKA1 can act as a co-regulator to induce the transcriptional repression of dual-specificity phosphatase 6 (DUSP6), facilitating EMT and tumor metastasis in ccRCC. Importantly, clinical data analysis revealed that double high expression of SKA1 combined with SAFB is a reliable indicator of a bad prognosis in people with ccRCC.

## RESULTS

### High SKA1 expression correlates with poor outcomes for cancer patients

**Figure 1:**
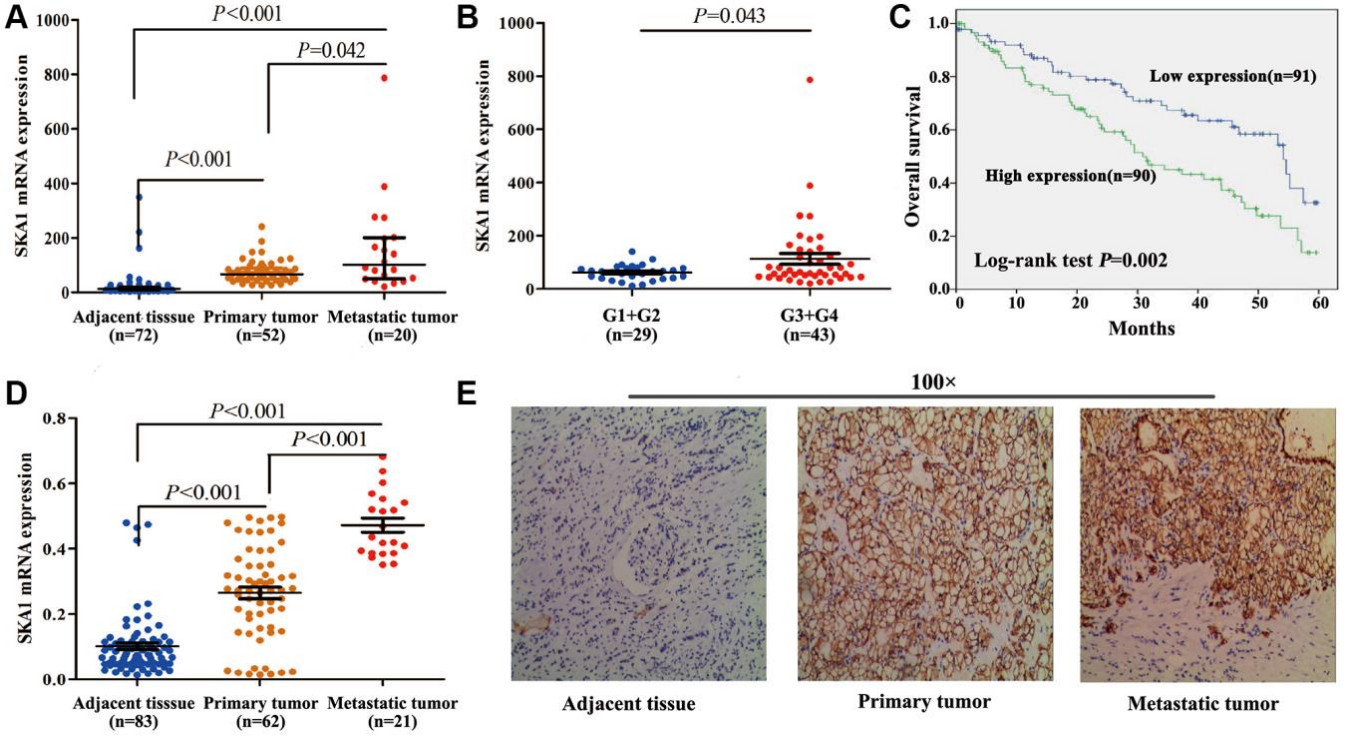
**High SKA1 expression positively correlates with human renal cancer progression.** (**A**–**C**), Analysis of SKA1 mRNA expression in ccRCC samples from TCGA-KIRC. (**A**) Analysis on SKA1 mRNA levels among ccRCC tissues, paired normal tissues, and metastatic tumor tissues (*N* = 72). (**B**) Analysis on SKA1 mRNA levels in low Fuhrman grade (G1+G2) and advanced-grade (G3+G4). (**C**) Kaplan–Meier analysis of ccRCC patient survival. (**D**) Analysis on SKA1 mRNA levels among ccRCC tissues, paired normal tissues, and metastatic tumor tissues (*N* = 83). (**E**) Representative images of IHC staining (magnification, ×100) for SKA1 protein from 83 ccRCC specimens.

SKA1 mRNA expression in ccRCC samples was evidently higher than in their corresponding normal counterparts, especially greater in metastatic tumor tissues than in normal and original tumor tissues, according to our initial analysis of the available clinical data sets TCGA (*N* = 72) (Figure 1A) [15]. Moreover, in comparison to that in ccRCC tissues at lower grade (G1+G2), the SKA1 level was strikingly higher in advanced-grade (G3+G4) ccRCC tissues (Figure 1B). Moreover, the elevated SKA1 levels were closely associated with Fuhrman grade, AJCC staging and malignancy metastasis (TCGA-KIRC, *N* = 248) (Supplementary Table 1A). Next, the TCGA-KIRC, *N* = 181 using a log-rank test, the Kaplan-Meier curve revealed that patients with high SKA1 expression had worse overall survival (Figure 1C). In addition, we used ccRCC patient datasets (TCGA-KIRC, *N* = 181) to perform univariate and multivariate regression analyses. According to univariate and multivariate analyses, the data revealed that SKA1 expression was an independent predictor for survival of ccRCC patients (Supplementary Table 1B). These findings imply, in brief, that ccRCC metastasis is facilitated by high SKA1 expression and that poor patient survival is correlated with high SKA1 expression.

To validate these results, we investigated SKA1 expression in 83 ccRCC specimens using immunohistochemistry (IHC). These patients were classified as high (score > 2) or low (score ≤ 2) SKA1 expression group according to the IHC scores. A pathological study revealed that increased SKA1 expression levels in ccRCC tissues were associated with a more aggressive tumor phenotype. (Supplementary Table 1C). Furthermore, qRT-PCR results showed that subjects with lymphatic metastasis had clearly higher levels of SKA1 mRNA expression than those without metastasis (Figure 1D). In addition, in most ccRCC tissues, SKA1 immunoreactive was primarily positive. (Figure 1E). Next, SKA1 expression was found in renal cancer cell lines. It turned out that in comparison to normal renal tubule epithelial cells HK-2, RCC cell lines 786-O, 769-P, ACHN, Caki-1, and OS-RC-2 displayed greater SKA1 mRNA and protein levels; nevertheless, endogenous SKA1 content did not significantly differ among these five renal cancer cell lines (Supplementary Figure 1A and 1B).

### Knockdown of SKA1 attenuates cancer metastasis *in vitro* and *in vivo*

**Figure 2:**
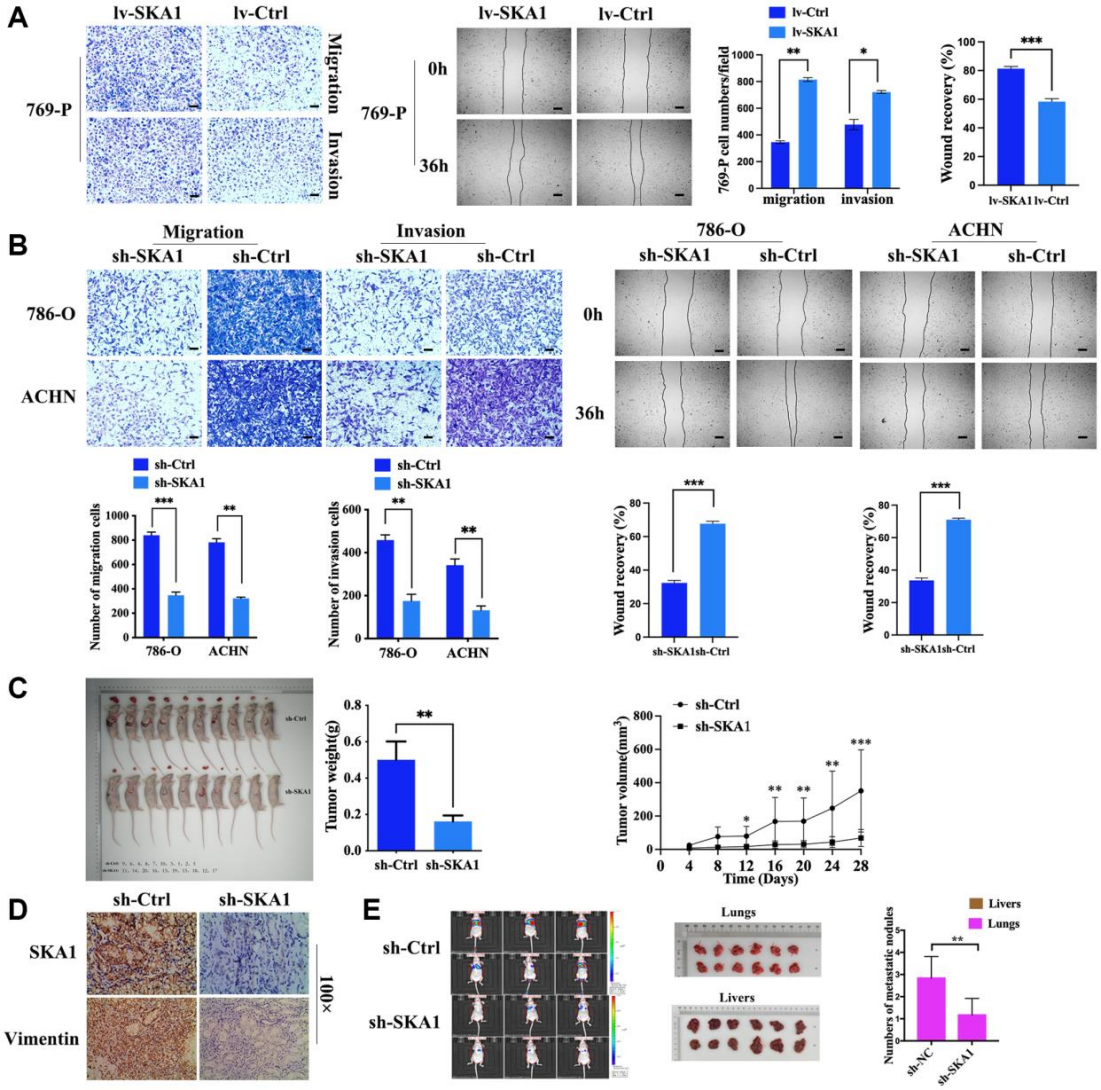
**Knockdown of SKA1 attenuates tumor cell motility *in vitro* and *in vivo*.** (**A** and **B**), Effects of SKA1 on the invasion and metastasis of RCC cells were confirmed by Transwell assay and wound healing assay. (**C**) The subcutaneous xenotransplantation model was established with 786-O cell line transfected with sh-SKA1 or sh-Ctrl. The length (L) and width (W) of tumors were measured once every four days for seven times after a six-week inoculation. At the end of investigation, mice were sacrificed and xenografts were then collected for diameter and weight measurements. (**D**) SKA1 and vimentin staining were performed in xenograft, scare bar: 100 μm. (**E**) Quantitation of tumor burdens determined by bioluminescence intensity, and numbers of metastatic nodules were counted in each group.

We selected 769-P to create cell lines that persistently overexpress SKA1 in order to probe further the oncogenic mechanism of SKA1 in ccRCC. Besides, 786-O and ACHN were chosen to construct the SKA1 stable knockdown cell line. Real-time PCR and western blot demonstrated SKA1 overexpression and knockdown (Supplementary Figure 2A and 2B). We discovered that upregulating SKA1 significantly improved the ability of 769-P cells to migrate and invade (Figure 2A). Correspondingly, RNA interference of SKA1 markedly decreased the mobility of tumor cells (Figure 2B). Apart from *in vitro* experiments, we used BALB/c mice (10 per group) for the subcutaneous tumor-bearing experiment and discovered that in comparison to the vector control group, the sh-SKA group’s tumor volume and weight were lower (*P* < 0.05). Furthermore, to determine if SKA1 can affect those malignant characteristics *in vivo*, a lung metastasis model was used. H&E staining results demonstrated that SKA1 knockdown in 786-O cells resulted in a significant reduction in the overall number of metastatic nodules in the lungs of nude mice (1.2 ± 0.8 nodes in the 786-O-sh-SKA1 group vs. 2.8 ± 1.0 nodes in the 786-O-sh-Ctrl group, *t* = 3.297, *p* = 0.008), although we didn’t find metastatic nodules in the liver of nude mice (Figure 2C and 2D). Consistently, lower SKA1 levels were detected in tumor sections derived from sh-SKA1-infected 786-O cells compared with their control counterparts. Immunostaining of vimentin (Figure 2E), a representative marker of cell mesenchymal phenotype, was weakened in tumor tissues from sh-SKA1-infected 786-O cells, which is consistent with *in vitro* results. Collectively, all these findings suggest that SKA1 has an apparent effect on the movement capacity of ccRCC cells.

### SKA1 silencing blocks TGF-β1-induced EMT in ccRCC cells

**Figure 3:**
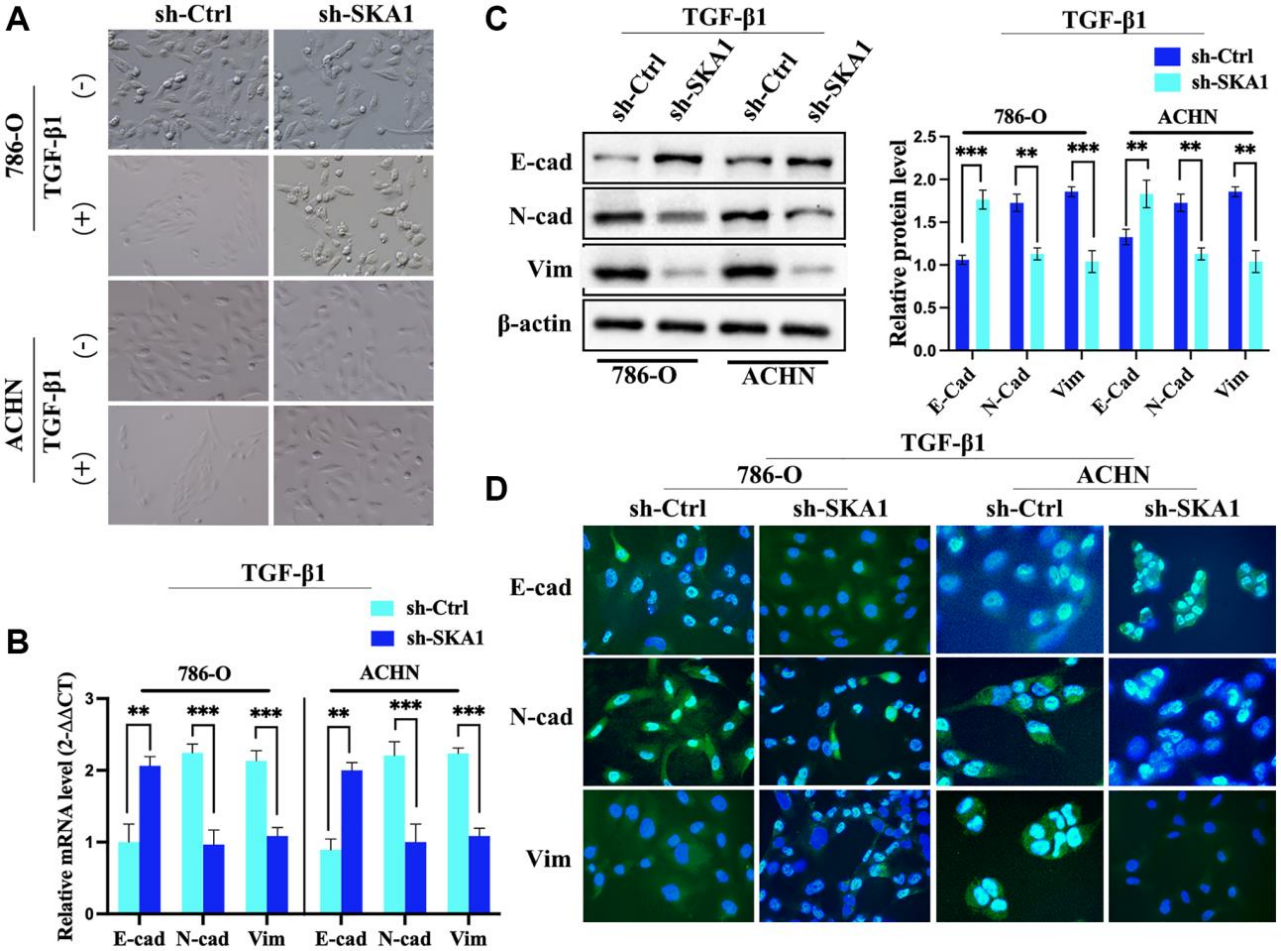
**SKA1 silencing blocks TGF-β1-induced EMT in ccRCC cells.** (**A**) Morphological analysis of 786-O and ACHN cells infected with control (sh-Ctrl) or SKA1 shRNA (sh-SKA1) with or without TGF-β1 treatment. Scale bar = 60 μm. (**B**) qPCR analysis of E-cadherin (E-cad) and vimentin mRNA levels in 786-O and ACHN cells infected with indicated constructs after TGF-β1 treatment. Mean fold change was calculated from three independent experiments performed in triplicate, with respect to sh-Ctrl-transfected cells artificially set by one. ^*^P < 0.05. (**C**) Western blot analysis of E-cad and vimentin protein levels in 786-O and ACHN cells. One representative experiment of three independent experiments are shown. Numbers indicate fold change in protein levels. (**D**) Immunofluorescence analysis of E-cad, N-cad and vimentin. Scale bar = 20 μm.

Erroneous EMT activation may result in tumor cell invasion, spread, and metastasis [16]. We examined the expression of EMT-related markers to learn more about SKA1’s function in the development of ccRCC. Control cells in a TGF-β1-induced EMT paradigm showed mesenchymal morphology after TGF-β1 treatment. In contrast, SKA1-depleted cells responded to TGF-β1 by maintaining their epithelial appearance (Figure 3A). The levels of genes connected to EMT were monitored for changes. In TGF-β1-treated ccRCC cells, lack of SKA1 increased the expression of the mesenchymal markers N-cadherin and vimentin while decreasing the amount of the epithelial marker E-cadherin, real-time PCR and western blot investigations (Figure 3B and 3C) reveal. Further confirmation of the elevation of E-cadherin together with a decrease in N-cadherin and vimentin in SKA1-depleted cells came from immunofluorescence analysis (Figure 3D). SKA1 is necessary for TGF-β1-induced EMT in ccRCC cells, to sum up.

### SKA1 was physically associated with SAFB in the nucleus

**Figure 4:**
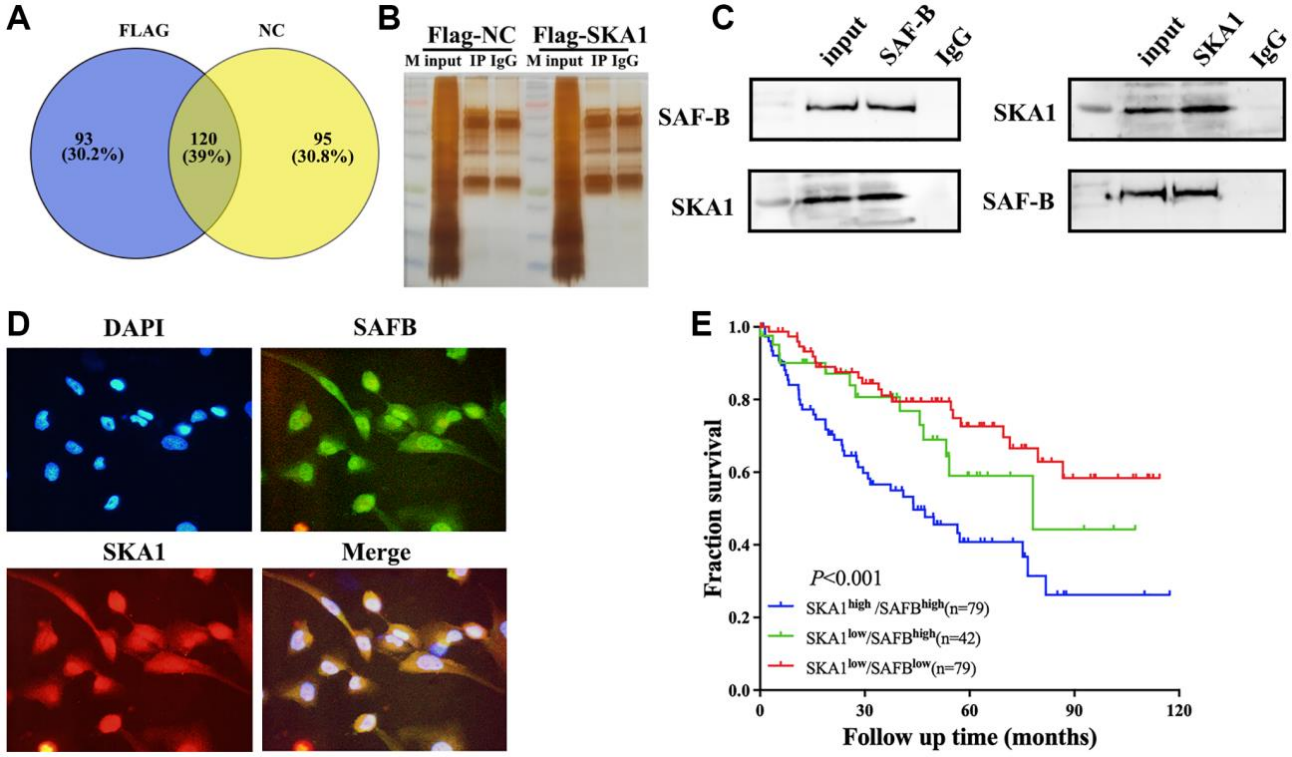
**SKA1 was physically associated with the SAFB in the nucleus.** (**A**) Pie diagram summarizing the SKA1-bound proteins as revealed by COIP- mass spectrometry. (**B** and **C**), Immunoprecipitation and mass spectrometry analysis of cofactors of SKA1. For the coimmunoprecipitation assay, normal IgG served as the negative control. (**D**) Confocal microscopy scan of immunofluorescence staining shows that SKA1 (green) co-localized with SAFB (red) in the 786-O cells. DAPI was used for nuclear staining. Scale bars = 10 μm. (**E**) Kaplan-Meier analysis of the overall survival in ccRCC patients from TCGA-KIRC (*N* = 242) according to the concurrent expression of SKA1 and SAFB.

Through its carboxy-terminal winged-helix domain, SKA1 engages in interactions with tubulin monomers, which explains how it is structurally supported in its ability to attach microtubule structures [17]. Additionally, SKA1 inhibits FPGS transcription by physically interacting with RPB3 to cause osteosarcoma cells to become resistant to MTX [18]. Thus, we reason that SKA1 might form a complex with interacting proteins that are instrumental in its functions in the ccRCC malignant process. To confirm this conjecture, endogenous SKA1-coupled proteins were screened through a coimmunoprecipitation (COIP) assay combined with mass spectrometry. In the SKA1 overexpression and vector control groups, respectively, proteomic analysis (uploaded as additional materials) found 93 and 95 proteins exclusive to the SKA1-related complexes, while 120 proteins were shared by both groups (Figure 4A). Supplementary Table 2 displays a list of the 93 distinct proteins that interact with SKA1. According to the scores and biological characteristics of SKA1 interacting proteins, we focused on SAFB, a nuclear matrix protein that connects to the scaffold or matrix attachment regions of the DNA elements, involved in processing essential for invasion and metastasis [19–21]. Reciprocal immunoprecipitation experiments demonstrated that SKA1 and SAFB interacted with each other in HEK-293T cells (Figure 4B and 4C), suggesting that SKA1 could form a complex with SAFB. Moreover, immunofluorescent staining showed that SKA1 was co-localized with SAFB in both the cytoplasm and nucleus, mainly in the nucleus (Figure 4D).

To dissect the question of whether SKA1 and SAFB play a clinical role and are implicated in the prognosis of renal carcinoma, aberrant expression of both genes was analyzed in the publicly available TCGA dataset consisting of 200 ccRCC patients with available follow-up data. The SKA1 and SAFB expression levels in the ccRCC tissues were used to classify all participants into three groups. Patients with double-high SKA1/SAFB expression appeared to have the worst 10-year survival rates, according to Kaplan-Meier analyses, 42 of 79 died in this subgroup (log-rank test, *P* < 0.001, hazard ratio = 2.330 [1.481–3.665]) (Figure 4E). Therefore, co-expression of SKA1 and SAFB predicted a worse prognosis in ccRCC patients.

### Gene expression analysis

**Figure 5:**
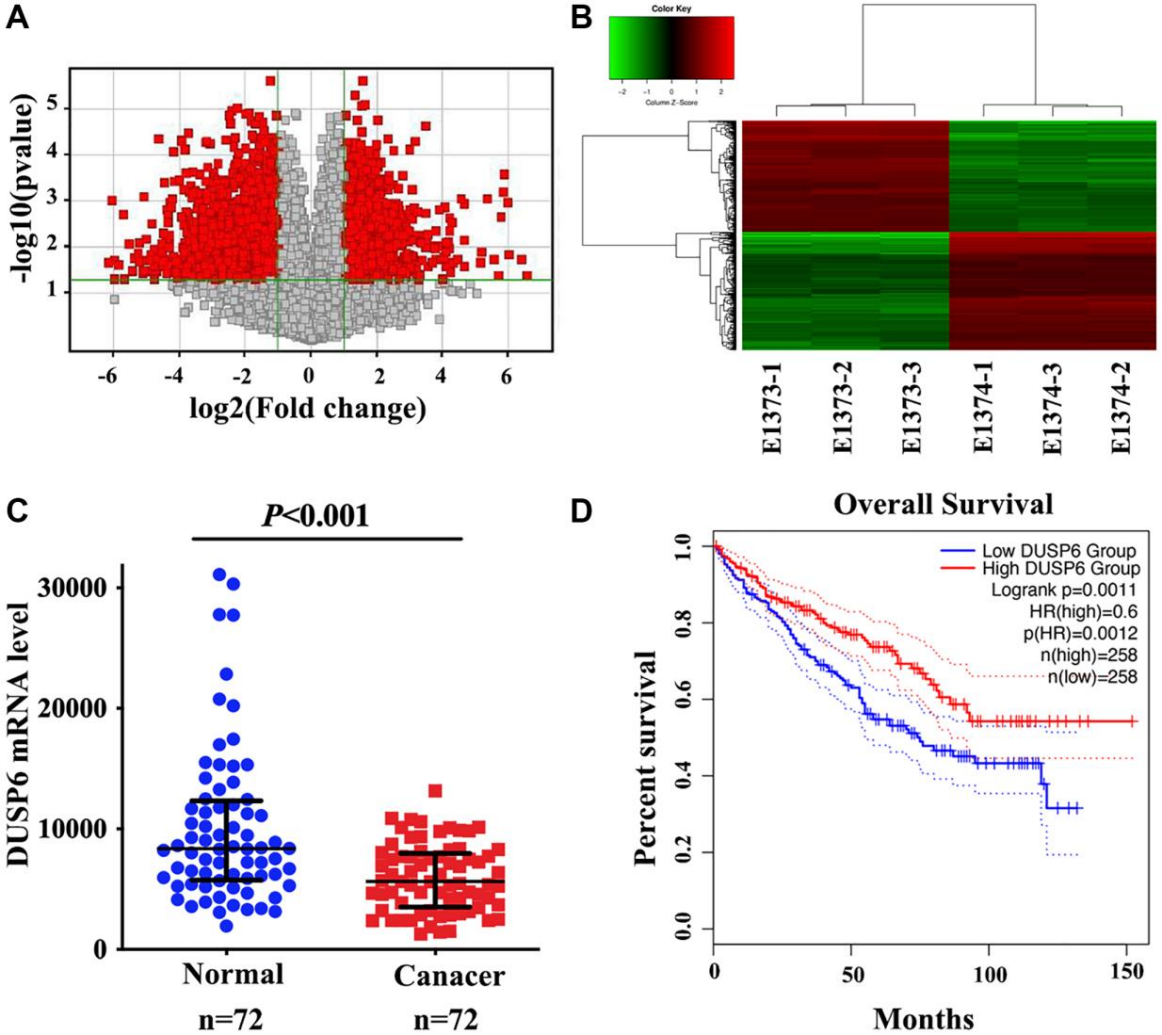
**Analysis of the mRNA profiles of SKA1 knockdown and control 786-O cells.** (**A**) Volcanic maps show the distribution of different genes between the sh-SKA1 and sh-Ctrl group. (**B**) Cluster diagrams show the aggregation of all samples and different genes at the expression level. (**C**) qRT-PCR analysis of DUSP6 mRNA expression in tumor and non-tumor renal tissues. (**D**) Kaplan-Meier curves for overall survival based on DUSP6 expression in ccRCC patients from TCGA-KIRC dataset (*N* = 516).

To better understand the variations in gene expression and probable causes of SKA1, we performed a genome-wide mRNA microarray study to compare the mRNA profiles of the sh-SKA1 and sh-Ctrl groups (Figure 5A and 5B). This gene microarray showed 535 differentially expressed gene transcripts (*P* < 0.05, Fold change ≧2.0) after data cleaning and filtering, when compared to the control group, 283 genes were upregulated and 252 genes were downregulated in the SKA1 silencing group (Supplementary Table 3A). Ingenuity Pathway Analysis (IPA) was utilized for relevant diseases and pathway analysis based upon differential expression genes. As shown in Supplementary Table 3B, GNRH signaling, known to be a crucial regulator in various types of malignancies [22–24], is the most significantly enriched pathway. The top five diseases and processes were angiogenesis, cell migration, cell movement, cell movement of tumor cell lines, and development of vasculature (Supplementary Table 3C). Additionally, the top 3 related networks encompassed organismal harm and abnormalities, gastrointestinal sickness, and cancer (Supplementary Table 3D). The MAPK phosphatase family member dual-specificity phosphatase 6 (DUSP6), may be a crucial effector for SKA1-mediated malignant progression of renal cell carcinoma based on the degree of connection between cancer cell motility and target genes, *p-*value, and fold change. A higher level of DUSP6 was observed in cases with abated expression of SKA1, and the converse is also true (Supplementary Figure 3A and 3B). To obtain pathological evidence, DUSP6 was first analyzed in the public ccRCC dataset from TCGA. It has been shown that DUSP6 was down-regulated compared to normal tissues in the ccRCC tissues (*n* = 72) (Figure 5C). Additionally, patients with low DUSP6 levels fared worse than those with high DUSP6 levels in terms of survival (*P* = 0.0011, obtained from the GEPIA, *N* = 516) (Figure 5D).

### DUSP6 mediates SKA1 depletion-induced tumor suppression *in vitro*

**Figure 6:**
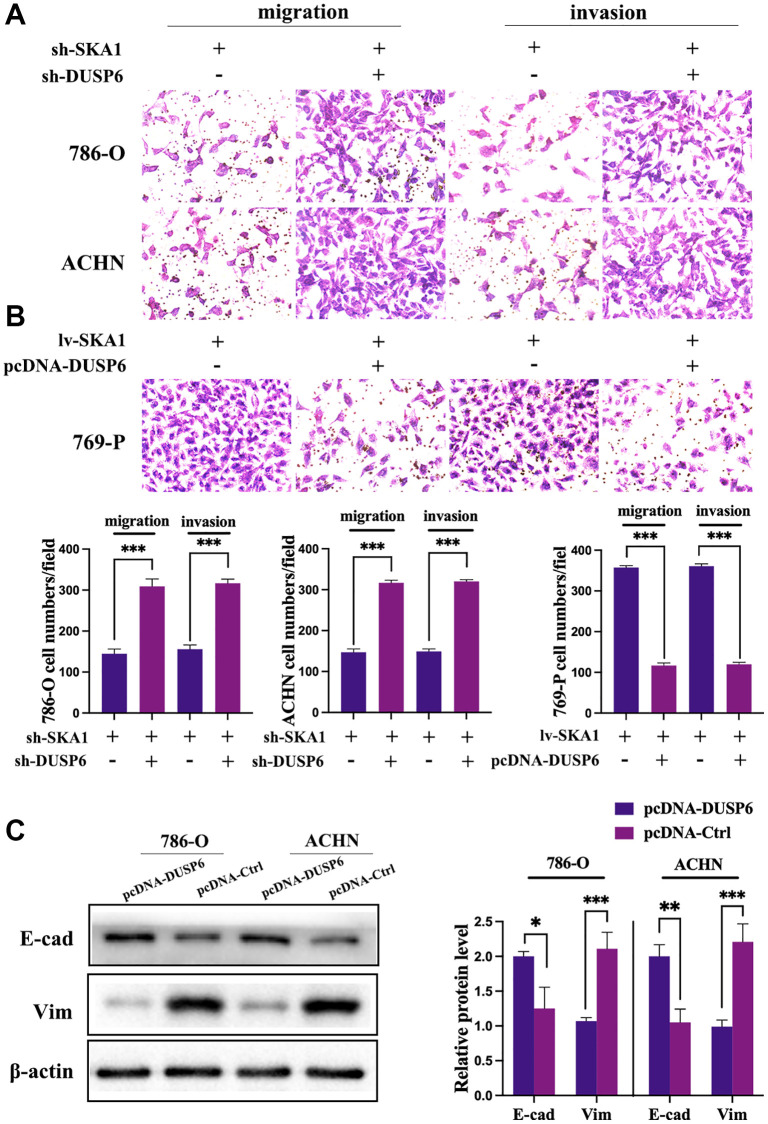
**DUSP6 mediates SKA1 depletion-induced tumor suppression.** (**A** and **B**), Transwell assay was used to assess the invasiveness and migration of cells transfected with indicated constructs. Data shown are mean from three independent experiments performed in triplicate. ^*^P < 0.05. (**C**) Western blot analysis of E-cadherin and vimentin protein levels in cells transfected with indicated constructs after TGF-β1 treatment. One representative experiment of three independent experiments are shown.

We came to the conclusion that since DUSP6 serves as a tumor suppressor in ccRCC, its overexpression may be to blame for the suppression of cancer cell migration and EMT brought on by SKA1 deficiency. The interference and overexpression efficiency of DUSP6 manipulated with the indicated plasmid was validated by qPCR and western blot (Supplementary Figure 4A and 4B). We discovered that DUSP6 knockdown restored the reduced invasive and migratory ability caused by SKA1 impairment (Figure 6A). DUSP6 expression plasmid co-infection, however, prevented the aggressive behavior brought on by SKA1 overexpression in ccRCC cells (Figure 6B). In both 786-O and ACHN cells, we also investigated DUSP6’s impact on variables associated with EMT. As seen in Figure 6C, in SKA1 over-expressed ccRCC cells, overexpression of DUSP6 lowered the quantity of vimentin and restored the amount of E-cadherin. The results suggested that SKA1-induced EMT was counteracted in DUSP6-positive cells. To sum up, these findings testify that SKA1’s ability to encourage the spread of cancer cells most likely works via suppressing DUSP6.

### SAFB is critical for the SKA1-mediated aggressive phenotype of ccRCC

**Figure 7:**
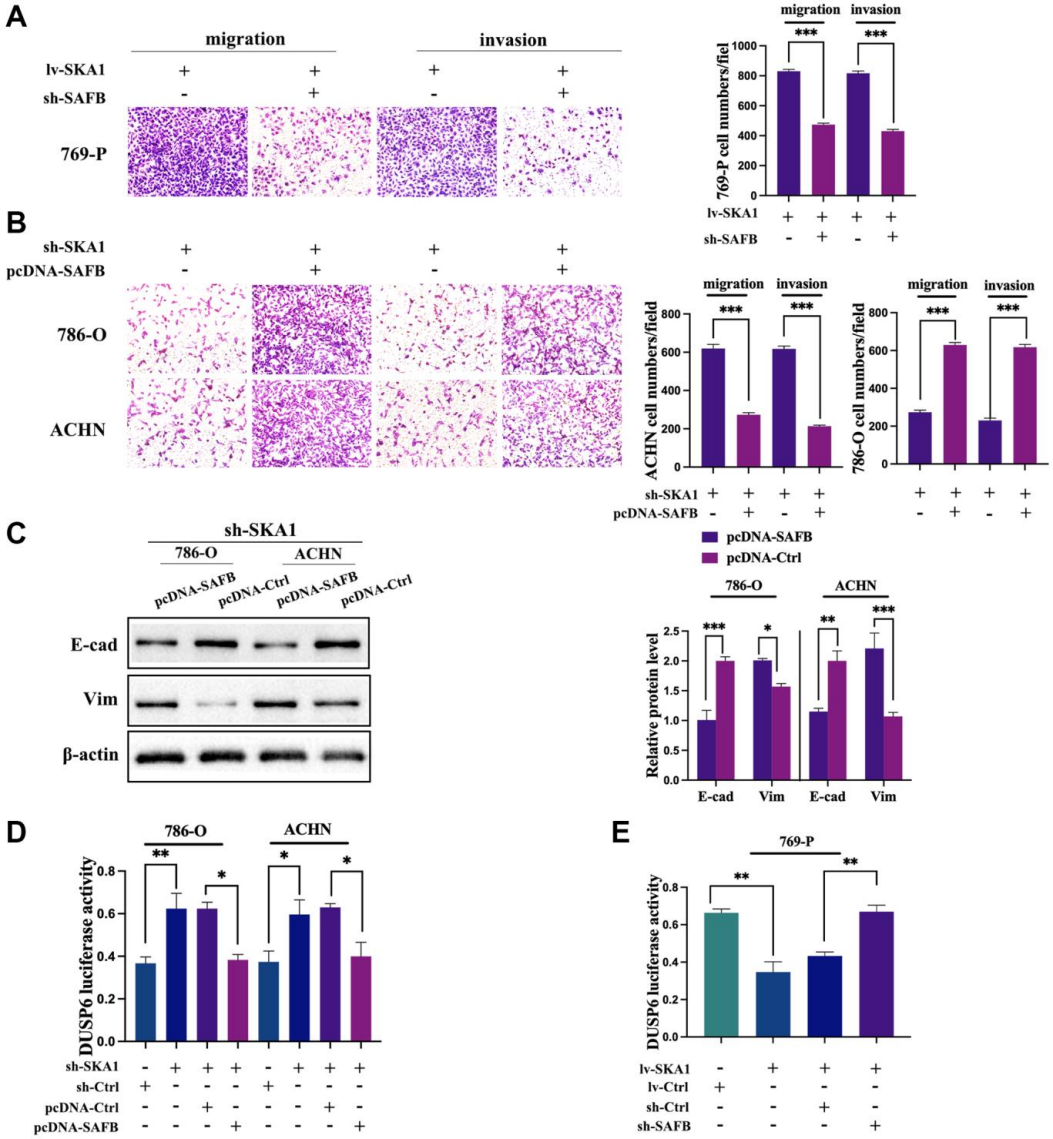
**SAFB is required for SKA1-mediated aggressive phenotype of ccRCC.** (**A**) Transwell assay was used to assess the invasiveness and migration of SKA1 overexpressing cells co-transfected siSAFB or scrambled control plasmid. (**B**) Transwell assay was used to assess the invasiveness and migration of SKA-depleted cells co-transfected SAFB overexpression or control plasmid. (**C**) Western blot analysis of E-cadherin and vimentin protein levels in cells transfected with indicated constructs after TGF-β1 treatment. One representative experiment of three independent experiments are shown. (**D**) Luciferase activity was determined in SKA-depleted cells co-transfected SAFB overexpression or control plasmid. (**E**) Luciferase activity was determined in SKA overexpressing cells co-transfected siSAFB or control plasmid. Data shown are mean from three independent experiments performed in triplicate. ^*^P < 0.05.

Next, we investigated SAFB’s potential involvement in the deterioration of ccRCC caused by SKA1. To assess whether SAFB was interfering with or overexpressing itself, qRT-PCR and western blot were performed (Supplementary Figure 5A and 5B). When endogenous SAFB was diminished, migration and invasive ability of 769-P cells induced by SKA1 overexpression were obviously attenuated (Figure 7A). Conversely, ectopic expression of SAFB retained the capability of migration and invasion in SKA1-depleted 786-O and ACHN cells (Figure 7B). Additionally, in SKA1-depleted cells, up-regulated SAFB resulted in a decrease in E-cadherin and an increase in vimentin (Figure 7C), indicating induction of EMT. These results imply that SAFB is essential for SKA1-mediated development of ccRCC malignancy.

Due to SAFB’s primary role in transcriptional repression, which is to impede the formation of transcriptional complexes [21], we ask if the downregulation of DUSP6 is a consequence of SAFB-mediated transcriptional repression in ccRCC cells. In this case, we carried out a luciferase test, and reporter activity was adjusted to the Renilla control. The results showed that knockdown of SKA1 brought about the enrichment of DUSP6 luciferase activity, while the effect was neutralized by co-infected SAFB (Figure 7D). Conversely, enforced SKA1 deeply inhibited luciferase activity, and the inhibitions could be reversed by co-transfection of sh-SAFB. (Figure 7E). Taken together, these data corroborate that SKA1 physically interplays with SAFB, which induces SAFB-dependent transrepression of DUSP6, consequently promoting cancer aggressiveness.

## DISCUSSION

Although multiple FDA-approved target medicines have resulted in breakthrough advancements, ccRCC patients still have a poor prognosis, with only 10% of them surviving after five years [25]. A critical first step in the creation of cutting-edge anticancer therapy strategies is comprehending the molecular underpinnings of metastatic renal carcinoma. In this research, we discovered that SKA1 is essential for tumor metastasis. Our bioinformatics and immunohistochemical investigations of clinical cancer patient materials revealed that increased SKA1 expression was associated with a poor outcome for patients with ccRCC, which provided initial evidence for this role. The SKA1 expression level was found to be an independent, substantial risk factor for overall survival of ccRCC following curative resection by univariate analysis. Additionally, *in vitro* and *in vivo* tests revealed that SKA1 successively speeds up the evolution of ccRCC and presumably aids cellular infiltration and dispersion by inducing EMT, an early stage in tumor metastasis [26, 27]. We demonstrate the notion using co-immunoprecipitation and co-localization analyses, and to the best of our knowledge, this is the first instance in which experimental proof of physical contact between SKA1 and SAFB has been presented. Additionally, we discovered that up-regulating SKA1 improved SAFB’s ability to bind to the DUSP6 promoter, which in turn reduced DUSP6 expression.

SKA1 was first discovered as a human mitotic spindle component in 2005 [28], and was later identified as one of the crucial elements needed for a timely anaphase beginning [9]. It has been reported that SKA1’s C-terminal domain serves as the principal microtubule-binding component of the SKA complex, and is essential for implementing biological functions [29]. SKA1 needs to act synergistically with Ndc80 to exert a pro-metastatic activity in the head and neck adenoid cystic carcinoma [30]. Moreover, SKA1 prevents RNA polymerase II subunit RPB3 from activating FPGS transcription by interacting with RPB3, consequently inducing MTX-resistance in osteosarcoma patients [18]. Of relevance here, our findings also indicate that SKA1 is mainly localized in the nucleus and interacts with scaffold attachment factor B (SAFB). Then, we show that SAFB can restore the invasive property in SKA1-depleted ccRCC cells and reverse EMT. So, we propose that SAFB is essential for the onset of SKA1-related illness. To find the precise mechanism for SKA1’s control of SAFB, more research is needed.

It is known that the translation of the SAFB genes results in big, multi-domain proteins with C-terminal sections that regulate the transcriptional repression activity of several genes [31]. SAFB was found to act as a transcriptional regulator, suppressing the expression of both genes by targeting the TATA box of the HSP27 promoter and the E-box of the hXOR promoter [32, 33]. Similar to this, SAFB slowed the development of colorectal cancer by concentrating on the TAK1 promoter’s initial E-box [21]. In this study, we conducted genome-wide mRNA microarray analysis and identified DUSP6 as a downstream target gene of SKA1. Functionally, we verified that expression of DUSP6 vitiates cell migration and EMT in SKA1 over-expressed ccRCC cells. Strong proof that SKA1 reduction could lessen SAFB binding to the DUSP6 promoter came from the luciferase experiment. On the other hand, SKA1 overexpression encouraged SAFB protein to bind to the DUSP6 promoter. These results support the hypothesis that SAFB transrepression activity is causally related to the downregulation of DUSP6 caused by SKA1. In clear cell renal cell carcinoma, our data convincingly demonstrated a mechanism by which SKA1 promotes tumor metastasis through SAFB-mediated transcriptional repression of DUSP6. However, additional research is necessary to fully understand the precise pattern by which SKA1 regulates SAFB.

Collectively, our data attest that SKA1 physically interacts with the SAFB and impels transcriptional repression of DUSP6, consequently provoking cancer aggressiveness. These results demonstrate the crucial role SKA1 plays in tumor spread and suggest that it may be a suitable therapeutic target for metastatic renal cancer.

## MATERIALS AND METHODS

### Clinical database

SKA1, SAFB, and DUSP6 expression levels in ccRCC patients were examined using the Cancer Genome Atlas (TCGA) database (https://cancergenome.nih.gov/). Downloaded from the TCGA were the datasets containing ccRCC patients with low and high levels of SKA1, SAFB, and DUSP6 mRNA expression, respectively, for survival comparisons and clinicopathological association studies. According to the TCGA publication standards (https://cancergenome.nih.gov/publications/publicationguidelines), the data processing was done correctly.

### Human specimens and cell lines

From January 2010 to December 2011, we enrolled 83 patients with pathologically diagnosed ccRCC at the Affiliated Tumor Hospital of Xinjiang Medical University. The Hospital Ethics Committee gave their approval to the study protocol. Each participant gave their written consent after receiving the necessary information. The date of the initial surgery was used to compute overall survival (OS), which was then calculated until either the tumor-related mortality occurred or the last follow-up date. None of the patients got any local or systemic treatment prior to surgery.

RCC cell lines (786-O, 769-P, ACHN, Caki-1 and OS-RC-2), normal renal tubule epithelial cells HK-2, and HEK293T cells were bought from the Cell Bank of the Chinese Academy of Sciences in Shanghai, China, and grown in DMEM or RPMI-1640 with 10% fetal bovine serum (FBS; Gibco, Grand Island, NY, USA). Cells were serum-starved for 12 hours before being treated for 24 hours with human recombinant TGF-1 (5 ng/mL; Calbiochem, San Diego, CA, USA) to induce EMT.

### Vector, shRNAs, and siRNAs

All plasmids and vectors were purchased from the OBIO Company (Shanghai, China). Two distinct shRNA sequences, SKA1 shRNA#1 and #2, and scrambled shRNA control were created and put into a lentiviral expression vector called GL427-pSLenti. Lentiviral expression vectors GL107-pSLenti were utilized for enforced expression of SKA1 and related vector control. The positive cells were selected by 10 μg/ml of puromycin, and the stable SKA1 expression cell line was confirmed by western bolt. SAFB or DUSP6 shRNA and scrambled control were designed and constructed into lentivirus plasmid Y6455-pLKD. Eukaryotic expression vector pcDNA3.1 (+)-MCS-3xFLAG was used for expression of SAFB or DUSP6 and related vector control. Transient expression of SAFB or DUSP6 was measured by qRT-PCR. The target sequences are as follows: sh1-SKA1 was 5′-GGAACAATTTGTCTAGT-3′, sh2-SKA1was 5′-GACATAAAGGAGTTAC-3′; sh1-SAFB was 5′-CTTCCGTGTCAGACCTTAAAG-3′, sh2-SAFB was 5′-GCGCTACCATTCTGACTTTAA-3′; sh1-DUSP6 was 5′-AAACTGTGGTG-TCTTGGTACAT-3′, sh2-DUSP6 was 5′-CTTGGACGTGTTGGAGGAATT-3′. The sequences of sh1-SKA1, sh1-SAFB and sh1-DUSP6 on account of stronger interference efficiency, would be used for follow-up research (Supplementary Figure 2C). Supplementary Table 4 of the Supplement contains a list of all primers.

### Protein extraction and Western blot

An ice-cold RIPA lysis solution with a complete protease inhibitor was used to extract the protein (Santa Cruz Biotechnology) and put it onto SDS-polyacrylamide gels for electrophoresis prior to transfer to nitrocellulose membranes. After inhibiting non-specific binding sites, particular antibodies were used to probe membranes. Primary antibodies for β-actin (sc-47778), SKA1 (ab91550), SAFB (ab187650), E-cadherin (ab40772), vimentin (ab92547) and DUSP6 (ab171765) were obtained from Abcam. Antibodies for SKA1 (PA5-61019) and SAFB (05-588) were purchased from Thermo Fisher and Sigma-Aldrich, respectively. Western blot secondary antibodies are Goat Anti-Mouse IgG H&L (HRP) (ab6789) and Goat Anti-Rabbit IgG H&L (HRP) (ab205718).

### Co-immunoprecipitation and mass spectrometry (CO-IP/MS)

SKA1-overexpressed and controlled HEK293T cells were lysed and precleared with anti-FLAG G-agarose beads and rabbit IgG. (14793s, Cell Signaling Technology). Immunoprecipitation of cellular lysates with anti-SKA1 or control IgG (Abcam, Cambridge, UK). Using anti-SKA1 or anti-SAFB antibodies, immunoprecipitated were examined using Western blotting.

The material was evaluated using an on-line nanospray LC-MS/MS on a Q Exactive™ Plus mass spectrometer (Thermo Fisher Scientific, MA, USA) coupled to an EASY-nanospray LC 1000 system (Thermo Fisher Scientific, MA, USA). PEAKS Studio X+ was utilized to process tandem mass spectra (Bioinformatics Solutions Inc., Waterloo, Canada). PEAKS DB was configured to search for uniprot homo sapiens (version201907, 20414 items) and customers provide a peptide database considering trypsin as the digesting enzyme. Peptides with -10lgP20 and proteins with -10lgP20 that contain at least one unique peptide were filtered.

### Luciferase reporter assay

The DUSP6 transcription start site’s long-range promoter was amplified and cloned into the pGL3 vector encoding the firefly luciferase reporter gene (ObiO, Shanghai, China). For the dual-luciferase gene reporter test, 786-O or ACHN cells were co-transfected with 0.1 mg of firefly luciferase constructs, 0.01 mg of pRL-TK renilla luciferase plasmid, and pcDNA3.1(+)-SAFB or pcDNA3.1(+)-control. Using a dual-luciferase gene reporter experiment, luciferase activity was assessed 48 hours after transfection (Promega, Madison, WI, USA). The results were calculated using relative luciferase activity (firefly luciferase/renilla luciferase).

### Gene microarray and data analysis

Total RNA was extracted from sh-SKA1 and control 786-O cells and hybridized to the Gene Chip Primeview Human gene array (901838; Affymetrix) using the GeneChip^®^ 3′ IVT Plus Kit (Affymetrix) in accordance with the manufacturer’s instructions. Ingenuity Pathway Analysis (IPA) (Ingenuity^®^ Systems, https://www.ingenuity.com) was used to build networks and functional analysis. The top 535 genes, illustrating the most significant changes due to the knockdown of SKA1, were submitted to the above website. Graphs illustrating gene expression based on SKA1 silencing were generated using STATA (StataCorp 2015, College Station, TX, USA).

### Immunofluorescence

Co-transfection of ACHN and 786-O cells with plasmids pCMV-3 × Flag-SKA1 and pcDNA3.1 (−)-myc-SAFB. The cells were then fixed with 4% paraformaldehyde and permeabilized with Triton X-100. For the detection of co-location, a mixture of SKA1 and SAFB antibodies was used. Coimmunostainings utilized donkey anti-rabbit IgG H&L/Alexa Fluor 594 and goat anti-mouse IgG H&L/FITC, and DAPI was employed to stain nuclei. An inverted fluorescent microscope was used to perform observations and take pictures.

### RNA extraction and qRT-PCR

Total RNA was extracted using the Trizol reagent (Invitrogen, Carlsbad, CA, USA) and reverse transcribed to cDNA using the Superscript III kit, all in accordance with the manufacturer’s instructions (Invitrogen). According to the manufacturer’s instructions, the resulting cDNA was amplified using SYBR Green-based qPCR (Sigma-Aldrich). A summary of PCR primers is provided in Supplementary Table 4. Using the 2^−ΔΔCT^ approach, the mRNA levels were standardized to that of glyceraldehyde 3-phosphate dehydrogenase (GAPDH).

### Immunohistochemistry and scoring

Anti-SKA1 antibodies were incubated with tissue slices (ab238716, Abcam, Cambridge, MA, USA). Without the primary antibody, the background staining was evaluated. In five areas of each sample, two skilled pathologists who were blinded to the clinical and pathologic information independently assessed the intensity and proportion of positive cells. The intensity of the stains was evaluated using a four-point scale (0, undetectable; 1, weak; 2, moderate; 3, strong). The percentage of positively stained cells was indicated as one of the four categories: 0, 0% to 25%; 2, 26% to 50%; 3, 51% to 75%; and 4, 76% to 100%. The expression of SKA1 was determined by multiplying the intensity and percentage scores.

### Transwell migration assay and invasion assay

Using a BD BioCoat™ Matrigel Invasion Chamber, a cell invasion test was conducted (BD Biosciences, San Jose, CA, USA). In 500 L of serum-free RPMI, 1 105 cells were grown and then seeded into the upper compartments of each chamber. For the lower compartments, 1 mL of RPMI with 10% FBS was introduced. After a 24-hour incubation at 37°C and 5% CO_2_, non-migrating and non-invading cells were scrubbed from the membrane’s upper surface. The cells on the flip side were stained with 0.1% crystal violet and counted at 100 magnification using a microscope.

### Wound healing assays

Six-well plates were planted with 786-O cells or ANCH cells. When cells attained confluence, a scratch wound was made using a 200–l plastic pipette tip. At 0 and 36 hours, the wound area was evaluated for its degree of contraction. Using Image J software, the size of each wound was determined.

### Subcutaneous xenotransplantation model

5 × 10^6^ 786-O cells stably transfected with sh-SKA1 or sh-Ctrl were injected subcutaneously into the right armpit of BALB/C female mice aged 4 weeks (10 mice per group). After a six-week inoculation, the length (L) and breadth (W) of tumors were measured every four days seven times. At the conclusion of the study, mice were slaughtered and xenografts were collected for measurements of diameter and weight. The expression level of (SKA1) and EMT (Vimentin) markers were evaluated using immunohistochemistry (IHC) testing. During the experiment, all animals were allocated into distinct groups using simple randomization.

### Lung metastasis assays

Twelve BALB/c nude mice (aged 4–5 weeks) were obtained from the Laboratory Animal Center at Xinjiang Medical University for this study. Randomly, the Mice were separated into two groups: the vector control group and the sh-SKA1 group. Two times 106 786-O-luc cells were injected into the tail vein of null mice. The IVIS Spectrum Imaging System was used to monitor lung metastases (Perkin Elmer Inc., Waltham, MA, USA). Six weeks after receiving an injection of cells, animals were slaughtered. Lung tissue was removed, fixed in 4% paraformaldehyde, and processed for histologic analysis.

### Statistical analysis

Using the χ2 test or Fisher’s exact test, the connection between the expression of SKA1 and other clinicopathological characteristics was investigated. Statistical studies between groups were conducted using the 2-tailed Student’s *t*-test or the Rank sum test, and the F Test was used to examine the variation between groups. To compare more than two groups, ANNOVA with post-hoc was conducted. The cumulative survival rate was computed using the Kaplan-Meier method and the log-rank test. Clinical factors that demonstrated a significant correlation with survival in both univariate and multivariate Cox proportional hazards model analyses. All statistical analyses were performed with SPSS 21 and GraphPad Prism 5, and *P* < 0.05 was regarded as statistically significant.

## Supplementary Materials

Supplementary Figures

Supplementary Tables 1A-1C and 4

Supplementary Table 2

Supplementary Table 3A

Supplementary Table 3B

Supplementary Table 3C

Supplementary Table 3D
