# Supplementary material for: SKA1 promotes tumor metastasis via SAFB-mediated transcription repression of DUSP6 in clear cell renal cell carcinoma
Source: Aging (Albany NY). 2022 Dec 2;14(23):9679–98. doi: 10.18632/aging.204418 (PMC9792197; doi:10.18632/aging.204418)
Supplement: Supplementary Figures [file aging-14-204418-s001.pdf]

## SUPPLEMENTARY FIGURES

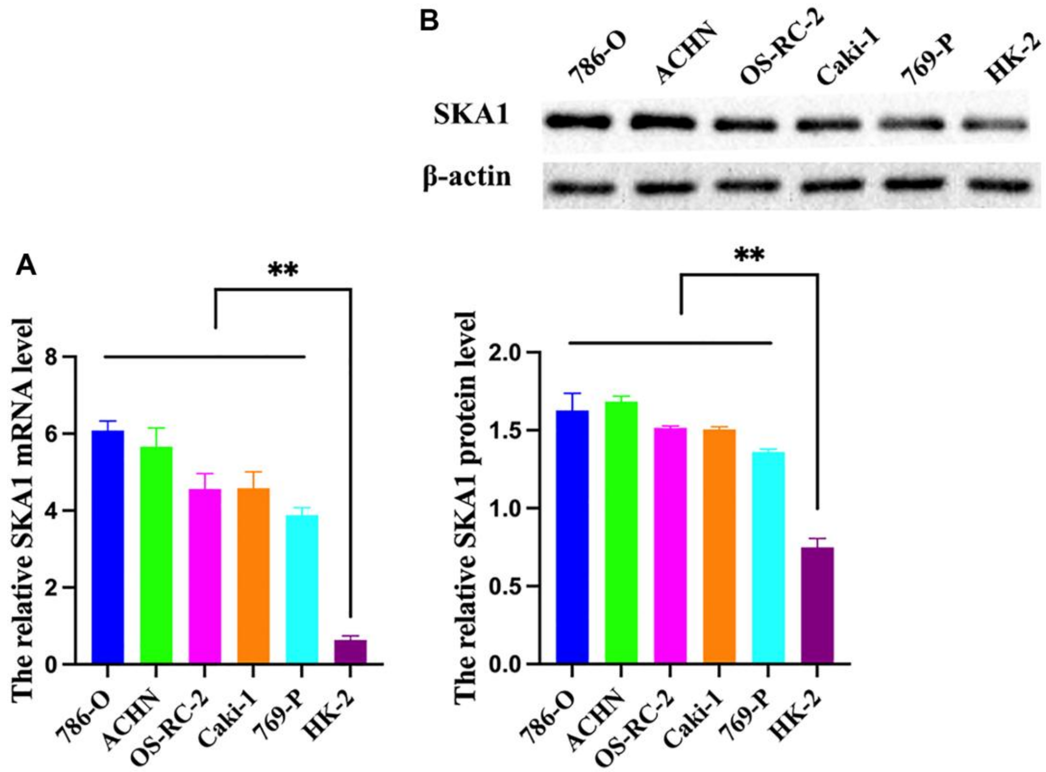


**Supplementary Figure 1.** (**A** and **B**) mRNA and protein expression level of SKA1 in a normal renal cell line (HK-2)and five ccRCC cell lines (786-O, 769-P, ACHN, Caki-1, and OS-RC-2).

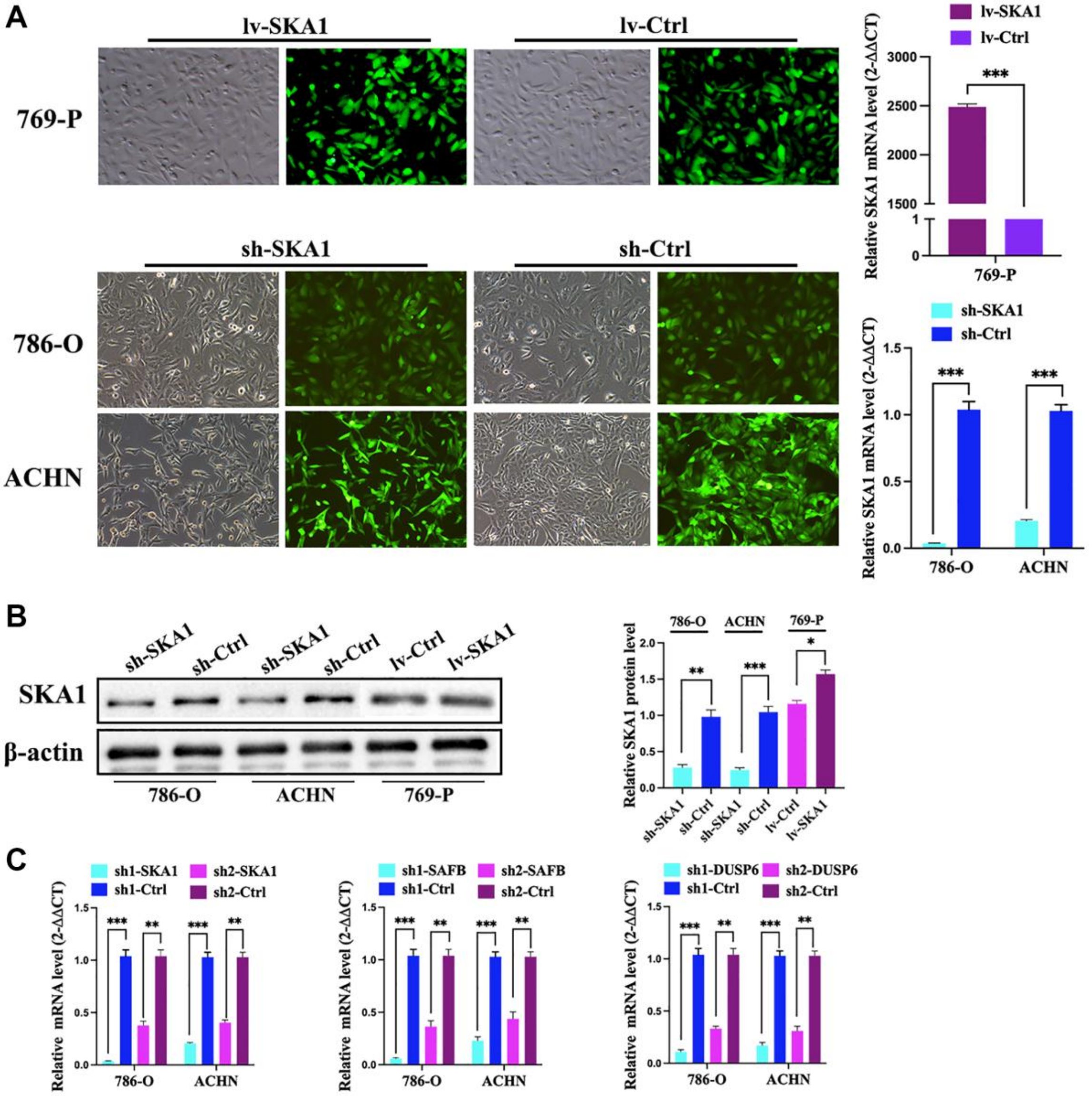


**Supplementary Figure 2.** (**A**–**C**) The overexpressing or knockdown efficiency of SKA1 was verified by qRT-PCR and Western blot assays in 769-P or 786-O and ACHN cells. (**C**) Comparison of the interference efficiency of two different sequences targeting SKA1, DUSP6 and SAFB genes, respectively.

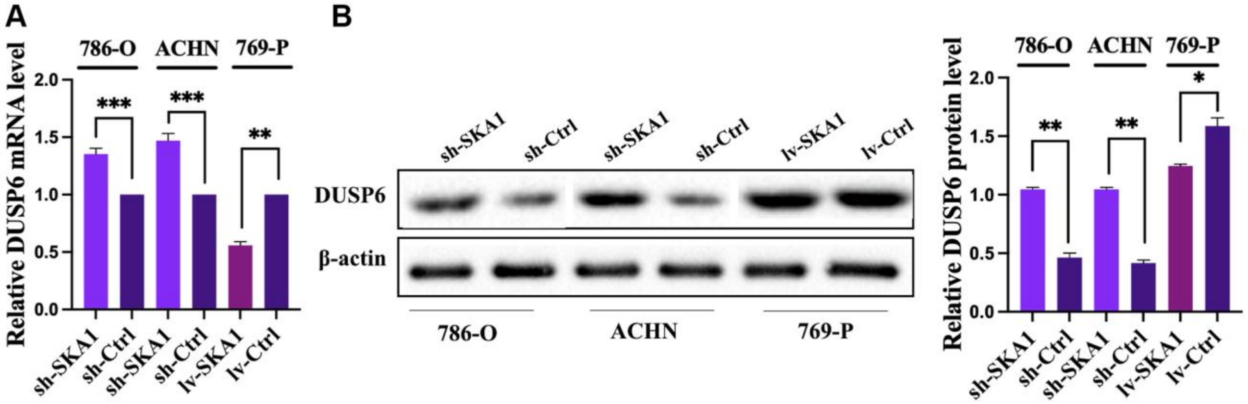


**Supplementary Figure 3.** (**A** and **B**) PCR and western blot analysis of DUSP6 mRNA and protein levels in 786-O, ACHN and 769-P cells.

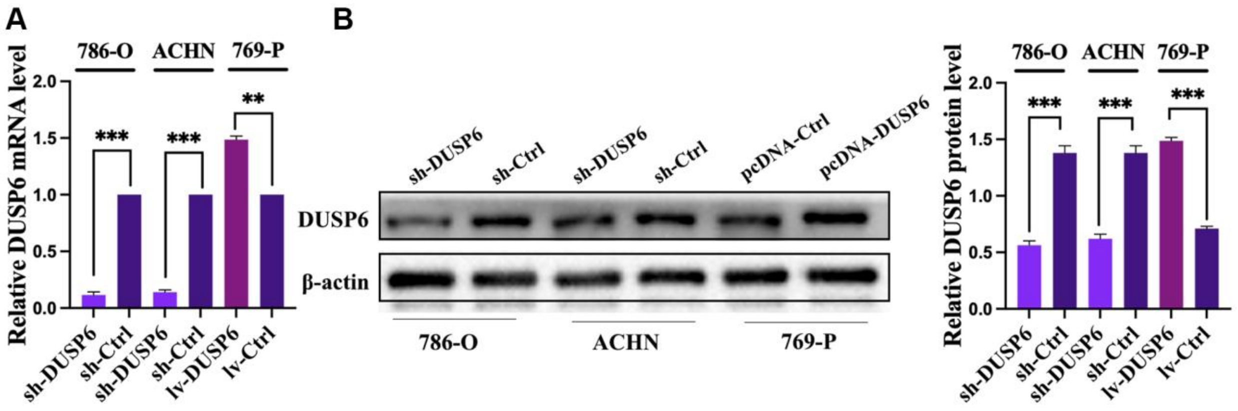


**Supplementary Figure 4.** (**A** and **B**) The interference and overexpression efficiency of DUSP6 manipulated with indicated plasmid was validated by qPCR and western blot.

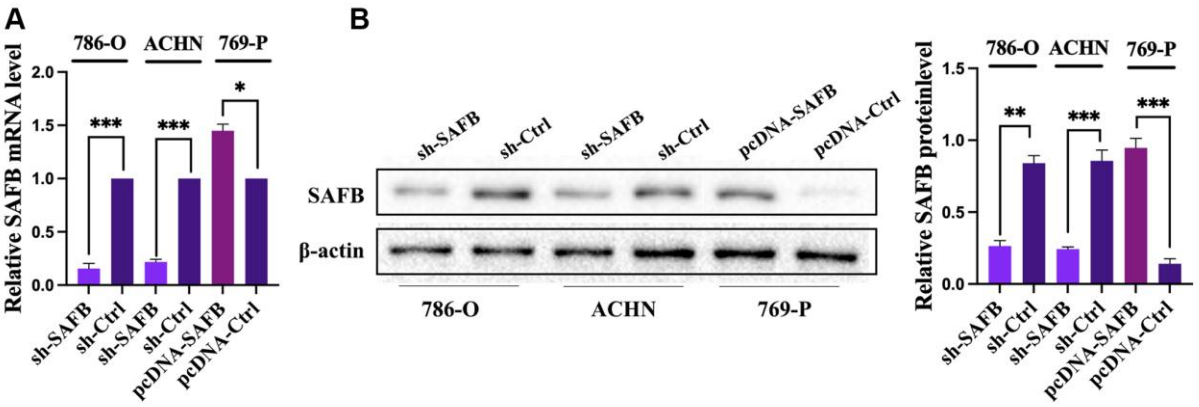


**Supplementary Figure 5.** (**A** and **B**) The interference and overexpression efficiency of SAFB manipulated with indicated plasmid was validated by qPCR and western blot.
