# Supplementary material for: SKA1 promotes tumor metastasis via SAFB-mediated transcription repression of DUSP6 in clear cell renal cell carcinoma
Source: Aging (Albany NY). 2022 Dec 2;14(23):9679–98. doi: 10.18632/aging.204418 (PMC9792197; doi:10.18632/aging.204418)
Supplement: Supplementary Tables 1A-1C and 4 [file aging-14-204418-s002.pdf]

Supplementary Tables

Supplementary Table 1A. Correlation between SKA1 expression and clinicopathological characteristics of ccRCC patients (*N* = 248) from TCGA-KIRC dataset.

Parameter	Number of cases	SKA1 mRNA expression		χ^2	*P*
		high expression (*n* = 124)	low expression (*n* = 124)		
Gender					
Male	150	82 (66.1)	68 (54.8)	3.307	0.069
Female	98	42 (33.9)	56 (45.2)		
Age					
<62	119	63 (50.8)	56 (45.2)	0.792	0.374
≥62	129	61 (49.2)	68 (54.8)		
Fuhrman grade					
G1+G2	111	38 (30.6)	73 (48.1)	19.978	<0.001
G3+G4	137	86 (69.4)	51 (58.9)		
AJCC stage					
I+II	135	52 (53.7)	83 (66.9)	16.623	<0.001
III+IV	113	72 (46.3)	41 (33.1)		
Lymphatic metastasis					
No	233	110 (88.7)	123 (99.2)	10.218	0.001
Yes	15	14 (11.3)	1 (0.8)		
Distant metastasis					
No	206	92 (74.2)	114 (91.9)	13.873	<0.001
Y es	42	32 (25.8)	10 (9.9)		

Supplementary Table 1B. Results of univariate and multivariate analysis for overall survival of ccRCC patients.

Risk factor	Univariate analysis			Multivariate analysis		
	HR*	*P* value	95% CI	HR*	*P* value	95% CI
Age	0.611	0.020*	0.403–0.927	0.584	0.014*	0.380–0.898
Gender	0.972	0.892	0.641–1.473	0.907	0.656	0.589–1.396
Fuhrman grade	0.398	<0.001***	0.253–0.625	0.636	0.075	0.387–1.047
AJCC stage	0.307	<0.001***	0.199–0.473	0.562	0.032*	0.332–0.952
Lymphatic metastasis	0.360	0.002**	0.186–0.695	0.637	0.205	0.317–1.280
Distant metastasis	0.262	<0.001***	0.170–0.403	0.439	0.002**	0.264–0.730
SKA1 Expression	0.470	<0.001***	0.309–0.715	0.770	0.273	0.482–1.229

Abbreviations: HR: Hazard Ratio; CI: Confidence interval.

Supplementary Table 1C. Correlation between SKA1 expression and clinicopathological characteristics of ccRCC patients (*N* = 83).

Clinicopathologic features	Number (*n* = 83)	SKA1 relative expression		χ^2	*P*
		high expression	low expression		
Age					
≤54	43	23	20	0.106	0.745
>54	40	19	21		
Gender					
Male	77	41	36	2.98	0.084
Female	6	1	5		

Tumor size (cm)					
≤5	35	15	20	0.966	0.326
>5	48	27	21		
AJCC stage					
I+II	51	32 19		6.594	0.010*
III+IV	32	10	22		
TNM stage					
T1+T2	48	25	23	0.009	0.752
T3+T4	35	17	18		
Fuhrman grade					
G1+G2	39	14	25	5.303	0.021*
G3+G4	44	28	16		
Lymph node metastasis					
Yes	8	2	6	1.326	0.249
No	75	40	35		
Distant metastasis					
Yes	15	11	4	4.196	0.041*
No	68	30	38		

Supplementary Table 4. The resultant cDNA was amplified by SYBR Green-based qPCR (Sigma-Aldrich) according to the manufacturer's instructions. Primer sequences for qPCR were displayed in the table.

Gene	Sequence (5′–3′)	Length (bp)
SKA1	F: ATGAAGAAACGAAGGATACCAAAG<br>R: CCTCGGACCTCTGATAGCC	142
DUSP6	F: CCCTGAGTACTAGCGTCCC<br>R: ACTCTACGATCAGGGTGGCC	131
SAFB	F: ATAGTCCTGTGAGTGCAGGCA<br>R: GTTCATAGGTTCCTCTACGCC	160
GAPDH	F: TGACTTCAACAGCGACACCCA<br>R: CACCCTGTTGCTGTAGCCAAA	121
