# Supplementary material for: SKA1 promotes tumor metastasis via SAFB-mediated transcription repression of DUSP6 in clear cell renal cell carcinoma
Source: Aging (Albany NY). 2022 Dec 2;14(23):9679–98. doi: 10.18632/aging.204418 (PMC9792197; doi:10.18632/aging.204418)
Supplement: Supplementary Table 2 [file aging-14-204418-s003.docx]

**Supplementary Table 2. The list of 93 unique proteins that interact with SKA1.**

| **Gene** | **Flag** | **Description** |
| --- | --- | --- |
| HBA1 | P69905 | Hemoglobin subunit alpha OS=Homo sapiens OX=9606 GN=HBA1 PE=1 SV=2 |
| SKA1 | Q96BD8 | Spindle and kinetochore-associated protein 1 OS=Homo sapiens OX=9606 GN=SKA1 PE=1 SV=1 |
| KRT6C | P48668 | Keratin type II cytoskeletal 6C OS=Homo sapiens OX=9606 GN=KRT6C PE=1 SV=3 |
| HNRNPA1 | P09651 | Heterogeneous nuclear ribonucleoprotein A1 OS=Homo sapiens OX=9606 GN=HNRNPA1 PE=1 SV=5 |
| SAFB | Q15424 | Scaffold attachment factor B1 OS=Homo sapiens OX=9606 GN=SAFB PE=1 SV=4 |
| CA1 | P00915 | Carbonic anhydrase 1 OS=Homo sapiens OX=9606 GN=CA1 PE=1 SV=2 |
| PRDX2 | P32119 | Peroxiredoxin-2 OS=Homo sapiens OX=9606 GN=PRDX2 PE=1 SV=5 |
| LYZ | P61626 | Lysozyme C OS=Homo sapiens OX=9606 GN=LYZ PE=1 SV=1 |
| KRT74 | Q7RTS7 | Keratin type II cytoskeletal 74 OS=Homo sapiens OX=9606 GN=KRT74 PE=1 SV=2 |
| RPS3A | P61247 | 40S ribosomal protein S3a OS=Homo sapiens OX=9606 GN=RPS3A PE=1 SV=2 |
| RPS2 | P15880 | 40S ribosomal protein S2 OS=Homo sapiens OX=9606 GN=RPS2 PE=1 SV=2 |
| TUBA1C | Q9BQE3 | Tubulin alpha-1C chain OS=Homo sapiens OX=9606 GN=TUBA1C PE=1 SV=1 |
| TUBA1A | Q71U36 | Tubulin alpha-1A chain OS=Homo sapiens OX=9606 GN=TUBA1A PE=1 SV=1 |
| TUBA4A | P68366 | Tubulin alpha-4A chain OS=Homo sapiens OX=9606 GN=TUBA4A PE=1 SV=1 |
| HNRNPH3 | P31942 | Heterogeneous nuclear ribonucleoprotein H3 OS=Homo sapiens OX=9606 GN=HNRNPH3 PE=1 SV=2 |
| IGHA1 | P01876 | Immunoglobulin heavy constant alpha 1 OS=Homo sapiens OX=9606 GN=IGHA1 PE=1 SV=2 |
| GLC2 | P0DOY2 | Immunoglobulin lambda constant 2 OS=Homo sapiens OX=9606 GN=IGLC2 PE=1 SV=1 |
| IGLC3 | P0DOY3 | Immunoglobulin lambda constant 3 OS=Homo sapiens OX=9606 GN=IGLC3 PE=1 SV=1 |
| RPS29 | P62273 | 40S ribosomal protein S29 OS=Homo sapiens OX=9606 GN=RPS29 PE=1 SV=2 |
| DEFA1 | P59665 | Neutrophil defensin 1 OS=Homo sapiens OX=9606 GN=DEFA1 PE=1 SV=1 |
| DEFA3 | P59666 | Neutrophil defensin 3 OS=Homo sapiens OX=9606 GN=DEFA3 PE=1 SV=1 |
| RPS19 | P39019 | 40S ribosomal protein S19 OS=Homo sapiens OX=9606 GN=RPS19 PE=1 SV=2 |
| RPS18 | P62269 | 40S ribosomal protein S18 OS=Homo sapiens OX=9606 GN=RPS18 PE=1 SV=3 |
| S100A8 | P05109 | Protein S100-A8 OS=Homo sapiens OX=9606 GN=S100A8 PE=1 SV=1 |
| PRDX1 | Q06830 | Peroxiredoxin-1 OS=Homo sapiens OX=9606 GN=PRDX1 PE=1 SV=1 |
| RPS11 | P62280 | 40S ribosomal protein S11 OS=Homo sapiens OX=9606 GN=RPS11 PE=1 SV=3 |
| CAT | P04040 | Catalase OS=Homo sapiens OX=9606 GN=CAT PE=1 SV=3 |
| CCT7 | Q99832 | T-complex protein 1 subunit eta OS=Homo sapiens OX=9606 GN=CCT7 PE=1 SV=2 |
| RPS25 | P62851 | 40S ribosomal protein S25 OS=Homo sapiens OX=9606 GN=RPS25 PE=1 SV=1 |
| RPL10 | P27635 | 60S ribosomal protein L10 OS=Homo sapiens OX=9606 GN=RPL10 PE=1 SV=4 |
| RPL10L | Q96L21 | 60S ribosomal protein L10-like OS=Homo sapiens OX=9606 GN=RPL10L PE=1 SV=3 |
| RPS14 | P62263 | 40S ribosomal protein S14 OS=Homo sapiens OX=9606 GN=RPS14 PE=1 SV=3 |
| LTF | P02788 | Lactotransferrin OS=Homo sapiens OX=9606 GN=LTF PE=1 SV=6 |
| LOR | P23490 | Loricrin OS=Homo sapiens OX=9606 GN=LOR PE=1 SV=2 |
| RPL28 | P46779 | 60S ribosomal protein L28 OS=Homo sapiens OX=9606 GN=RPL28 PE=1 SV=3 |
| GRN | P28799 | Progranulin OS=Homo sapiens OX=9606 GN=GRN PE=1 SV=2 |
| SLIRP | Q9GZT3 | SRA stem-loop-interacting RNA-binding protein mitochondrial OS=Homo sapiens OX=9606 GN=SLIRP PE=1 SV=1 |
| SPRR2B | P35325 | Small proline-rich protein 2B OS=Homo sapiens OX=9606 GN=SPRR2B PE=2 SV=1 |
| SPRR2D | P22532 | Small proline-rich protein 2D OS=Homo sapiens OX=9606 GN=SPRR2D PE=2 SV=2 |
| SPRR2E | P22531 | Small proline-rich protein 2E OS=Homo sapiens OX=9606 GN=SPRR2E PE=2 SV=2 |
| SPRR2F | Q96RM1 | Small proline-rich protein 2F OS=Homo sapiens OX=9606 GN=SPRR2F PE=3 SV=1 |
| SPRR2A | P35326 | Small proline-rich protein 2A OS=Homo sapiens OX=9606 GN=SPRR2A PE=1 SV=1 |
| SPRR2G | Q9BYE4 | Small proline-rich protein 2G OS=Homo sapiens OX=9606 GN=SPRR2G PE=3 SV=1 |
| RPL36A | P83881 | 60S ribosomal protein L36a OS=Homo sapiens OX=9606 GN=RPL36A PE=1 SV=2 |
| RPL36AL | Q969Q0 | 60S ribosomal protein L36a-like OS=Homo sapiens OX=9606 GN=RPL36AL PE=1 SV=3 |
| IGKC | P01834 | Immunoglobulin kappa constant OS=Homo sapiens OX=9606 GN=IGKC PE=1 SV=2 |
| RPL23 | P62829 | 60S ribosomal protein L23 OS=Homo sapiens OX=9606 GN=RPL23 PE=1 SV=1 |
| HIST3H2BB | Q8N257 | Histone H2B type 3-B OS=Homo sapiens OX=9606 GN=HIST3H2BB PE=1 SV=3 |
| HIST1H2BA | Q96A08 | Histone H2B type 1-A OS=Homo sapiens OX=9606 GN=HIST1H2BA PE=1 SV=3 |
| HIST2H2BE | Q16778 | Histone H2B type 2-E OS=Homo sapiens OX=9606 GN=HIST2H2BE PE=1 SV=3 |
| HIST1H2BJ | P06899 | Histone H2B type 1-J OS=Homo sapiens OX=9606 GN=HIST1H2BJ PE=1 SV=3 |
| HIST1H2BB | P33778 | Histone H2B type 1-B OS=Homo sapiens OX=9606 GN=HIST1H2BB PE=1 SV=2 |
| HIST1H2BO | P23527 | Histone H2B type 1-O OS=Homo sapiens OX=9606 GN=HIST1H2BO PE=1 SV=3 |
| IGLV3-19 | P01714 | Immunoglobulin lambda variable 3-19 OS=Homo sapiens OX=9606 GN=IGLV3-19 PE=1 SV=2 |
| BLVRB | P30043 | Flavin reductase (NADPH) OS=Homo sapiens OX=9606 GN=BLVRB PE=1 SV=3 |
| IGKV2-28 | A0A075B6P5 | Immunoglobulin kappa variable 2-28 OS=Homo sapiens OX=9606 GN=IGKV2-28 PE=3 SV=1 |
| IGKV2D-28 | P01615 | Immunoglobulin kappa variable 2D-28 OS=Homo sapiens OX=9606 GN=IGKV2D-28 PE=1 SV=2 |
| IGKV2-40 | A0A087WW87 | Immunoglobulin kappa variable 2-40 OS=Homo sapiens OX=9606 GN=IGKV2-40 PE=3 SV=2 |
| IGKV2D-40 | P01614 | Immunoglobulin kappa variable 2D-40 OS=Homo sapiens OX=9606 GN=IGKV2D-40 PE=1 SV=2 |
| CCT6A | P40227 | T-complex protein 1 subunit zeta OS=Homo sapiens OX=9606 GN=CCT6A PE=1 SV=3 |
| SLC4A1 | P02730 | Band 3 anion transport protein OS=Homo sapiens OX=9606 GN=SLC4A1 PE=1 SV=3 |
| EIF4A2 | Q14240 | Eukaryotic initiation factor 4A-II OS=Homo sapiens OX=9606 GN=EIF4A2 PE=1 SV=2 |
| RPL8 | P62917 | 60S ribosomal protein L8 OS=Homo sapiens OX=9606 GN=RPL8 PE=1 SV=2 |
| RPL39P5 | Q59GN2 | Putative 60S ribosomal protein L39-like 5 OS=Homo sapiens OX=9606 GN=RPL39P5 PE=5 SV=2 |
| RPL39 | P62891 | 60S ribosomal protein L39 OS=Homo sapiens OX=9606 GN=RPL39 PE=1 SV=2 |
| PCMT1 | P22061 | Protein-L-isoaspartate(D-aspartate) O-methyltransferase OS=Homo sapiens OX=9606 GN=PCMT1 PE=1 SV=4 |
| RPL24 | P83731 | 60S ribosomal protein L24 OS=Homo sapiens OX=9606 GN=RPL24 PE=1 SV=1 |
| RPS15 | P62841 | 40S ribosomal protein S15 OS=Homo sapiens OX=9606 GN=RPS15 PE=1 SV=2 |
| SETD2 | Q9BYW2 | Histone-lysine N-methyltransferase SETD2 OS=Homo sapiens OX=9606 GN=SETD2 PE=1 SV=3 |
| SLPI | P03973 | Antileukoproteinase OS=Homo sapiens OX=9606 GN=SLPI PE=1 SV=2 |
| SHROOM3 | Q8TF72 | Protein Shroom3 OS=Homo sapiens OX=9606 GN=SHROOM3 PE=1 SV=2 |
| PTPRO | Q16827 | Receptor-type tyrosine-protein phosphatase O OS=Homo sapiens OX=9606 GN=PTPRO PE=1 SV=2 |
| IGKV4-1 | P06312 | Immunoglobulin kappa variable 4-1 OS=Homo sapiens OX=9606 GN=IGKV4-1 PE=1 SV=1 |
| RPL35A | P18077 | 60S ribosomal protein L35a OS=Homo sapiens OX=9606 GN=RPL35A PE=1 SV=2 |
| THRAP3 | Q9Y2W1 | Thyroid hormone receptor-associated protein 3 OS=Homo sapiens OX=9606 GN=THRAP3 PE=1 SV=2 |
| RTCB | Q9Y3I0 | tRNA-splicing ligase RtcB homolog OS=Homo sapiens OX=9606 GN=RTCB PE=1 SV=1 |
| HIBCH | Q6NVY1 | 3-hydroxyisobutyryl-CoA hydrolase mitochondrial OS=Homo sapiens OX=9606 GN=HIBCH PE=1 SV=2 |
| SUPT5H | O00267 | Transcription elongation factor SPT5 OS=Homo sapiens OX=9606 GN=SUPT5H PE=1 SV=1 |
| MRPS14 | O60783 | 28S ribosomal protein S14 mitochondrial OS=Homo sapiens OX=9606 GN=MRPS14 PE=1 SV=1 |
| NUTM2G | Q5VZR2 | NUT family member 2G OS=Homo sapiens OX=9606 GN=NUTM2G PE=3 SV=2 |
| NUTM2F | A1L443 | NUT family member 2F OS=Homo sapiens OX=9606 GN=NUTM2F PE=2 SV=2 |
| NUTM2D | Q5VT03 | NUT family member 2D OS=Homo sapiens OX=9606 GN=NUTM2D PE=3 SV=2 |
| NUTM2E | B1AL46 | NUT family member 2E OS=Homo sapiens OX=9606 GN=NUTM2E PE=3 SV=3 |
| NUTM2B | A6NNL0 | NUT family member 2B OS=Homo sapiens OX=9606 GN=NUTM2B PE=3 SV=2 |
| NUTM2A | Q8IVF1 | NUT family member 2A OS=Homo sapiens OX=9606 GN=NUTM2A PE=2 SV=4 |
| HELZ | P42694 | Probable helicase with zinc finger domain OS=Homo sapiens OX=9606 GN=HELZ PE=1 SV=2 |
| ZNF831 | Q5JPB2 | Zinc finger protein 831 OS=Homo sapiens OX=9606 GN=ZNF831 PE=2 SV=4 |
| MLLT3 | P42568 | Protein AF-9 OS=Homo sapiens OX=9606 GN=MLLT3 PE=1 SV=2 |
| MICAL3 | Q7RTP6 | [F-actin]-monooxygenase MICAL3 OS=Homo sapiens OX=9606 GN=MICAL3 PE=1 SV=2 |
| CDSN | Q15517 | Corneodesmosin OS=Homo sapiens OX=9606 GN=CDSN PE=1 SV=3 |
| AZGP1 | P25311 | Zinc-alpha-2-glycoprotein OS=Homo sapiens OX=9606 GN=AZGP1 PE=1 SV=2 |
| RPL19 | P84098 | 60S ribosomal protein L19 OS=Homo sapiens OX=9606 GN=RPL19 PE=1 SV=1 |
| TGM3 | Q08188 | Protein-glutamine gamma-glutamyltransferase E OS=Homo sapiens OX=9606 GN=TGM3 PE=1 SV=4 |
